# Collectanea Medica: Consisting of Anecdotes, Facts, Extracts, Illustrations, &c.

**Published:** 1826-02

**Authors:** 


### COLLECTANEA MEDICA:

CONSISTING OF

### ANECDOTES, FACTS, EXTRACTS, ILLUSTRATIONS, &c.

***Relating to the History or the Art of Medicine, and the Collateral Sciences.***

Floriferus, ut apes, in saltibus omnia libant,
Omnia nos, itidem, depascimur aurea dicta.

ART. I.—*Note on the Actions of the Muscular System.* By JOHN D. GODMAN, M.D. [From the *Philadelphia Journal,* No. 2, New Series.]

DURING a great lapse of time, the muscular system has been carefully studied by numerous intelligent and acute observers, in all countries where science has been cultivated; yet, notwithstanding their important labours, much remains to be known and explained relative to the structure of the muscles, and their modes of action. It is not with an expectation of supplying what is wanting to complete our knowledge of the muscular system that this note is written, but to invite attention to some interesting particulars which have been singularly overlooked by those who have heretofore treated of muscular action.

However rough and irregular the skeleton may appear when entirely denuded, it is most admirably formed for giving support and proper attachments to the soft parts designed for the growth and motion of the frame; and, in the living condition, the adaptation of the soft textures to the bony fabric is productive of every variety of beautiful outline, whether the body be in a state of exertion or of repose.

In various parts of the system, beauty of configuration is obtained at the expense of power: the motive instrument is not placed in a situation where it can exert the greatest possible degree of force, neither is the lever it operates on the most advantageous for raising a given weight: still the effect is produced with the least sacrifice of convenience and beauty, and all apparent disadvantages are fully compensated by the combined action of different muscles. This is very evident when we reflect that the lever of the third order is the one most univerally employed in the human body; the lever itself being the least efficient of all, yet allowing an arrangement of muscles, &c. about the bone, the most conducive to symmetry and convenience; while the want of absolute power from the use of this lever is compensated by the greater number of muscles brought into action, and the much greater variety of motions to be performed, in consequence of the peculiar relations of the bones.

In addition to the general attention to symmetrical arrangement displayed in the muscular system, there are very numerous instances of wonderful design exhibited in the combinations effected for the purpose of modifying and directing muscular action; and to these I more especially wish at present to refer. The study of these modifying causes opens a wide field of observation to the physiologist and rational anatomist, and impart to the muscular system a degree of interest sufficient to repay one for all the labour endured in gaining an acquaintance with its minutiæ.

It has, no doubt, forced itself on the mind of every one who has attempted the study of anatomy, that the mere enunciation of a muscle arising at one point and being fixed to another, is, to say the least, a very dry and uninviting task for the memory. When such an enunciation is coupled with an apothegmatic sentence, declaring the use of the muscle, as a general rule, it is received as a thing to be believed because it is said, rather than as a proper consequence of the origin and insertion before stated. There is a great deal wanting to the establishment of a proper conclusion in the mind of the learner, and for a good reason,—the action ascribed to three-fourths of the muscles could not possibly take place, if it were not for the peculiar causes which modify and direct the exertion of their powers. These, both in books and public lectures, are as entirely left out of sight as if they did not exist, or rather as if they were utterly unknown to writers and teachers. The latter inference may be considered fair; for we believe that no man, knowing the circumstances, would pass them by in silence.

The circumstances modifying muscular action have been my favourite study for some years past: they have led to all the observations relative to the fasciæ, heretofore published in this Journal, and to all the discoveries made known relative to the capsular ligaments of joints, &c. in my Anatomical Investigations. I shall at this time take a view of the modifiers of muscular action, under the following heads:—

1st. Fasciæ and sheaths.

2d. Position in regard to bones; relation of muscular fibres to tendons.

3d. Modifying muscles.

4th. Modifying tendinous connexions.

5th. Special modifying constructions; annular ligaments, trochleæ, &c.

1st. *Fasciæ and sheaths.*—The fasciæ covering the extremities of the body will be the fairest exemplification of this part of our subject, because they are the most obvious and generally known. They are strong, dense, and inelastic fibrous membranes, stretching from the bones over the muscles, so as to give them an uniform external covering; and thus far the fasciæ were studied previous to my researches. In addition to the external covering, sheets or layers are sent off from the great exterior sheet, by which each of the muscles is enwrapped or included in a distinct sheath, the layers of which terminate on and around the joints for the formation of their capsular ligaments. In consequence, the muscles thus covered, instead of swelling during their contraction in a single mass, resisted only by the general external covering, contract and swell within their own particular sheaths besides; and these, being fixed to the bone as well as to the external fasciæ, direct the force of the muscle to the greatest possible advantage. The same circumstance of the sheaths for the individual muscles being formed from the great common fasciæ, enables us to understand how muscles, apparently very similar in place and appearance, are capable of performing very different actions.

Let us, to make the idea clearer, consider some of the muscles individually. The *sartorius* arises from the anterior superior spine of the ilium, and is inserted into the tuber of the tibia, its use being to cross the legs on each other, as is done by tailors when seated at work; whence the muscle has its name. But the sartorius is the longest muscle in the human body: its origin is very nearly over the median line of each thigh; to reach its insertion, it passes under the inside of the knee to arrive at the tibia. When the muscle contracts, it makes an effort to straighten itself; and, if it were not forced by some cause to contract in the line corresponding to its course while in a state of repose, it could not produce the movement above mentioned. What is this necessary modifying power? It cannot be the general or external fascia lata; because, if this were all, it might compress the muscle during its contraction, but could not prevent it from being drawn towards the middle of the thigh, or even from starting over the inner condyle of the femur. On examination, we readily discover the modifying cause to be the sheath, or double layer of fascia, in which this muscle is included; and this, together with the general fascia, so binds it to its place as to prevent it from starting in any direction, or producing inconvenience or deformity. The efficacy of the sheath is distinctly manifest in this case, but not more so than it is in all the muscles belonging to the extremities.

There are a few instances where muscles have their actions modified by the agency of a single sheet of fascia on their exterior, though in these cases the term *aponeurosis* is most applicable to the modifying membrane. Such is the aponeurosis of the temporal muscle, the palmar and plantar aponeurosis, which are the only parts not belonging to the great general fasciæ of the body. The mass of the temporal muscle arises on the side of the skull, but a layer of muscular fibre, concerned in modifying the action of the whole muscle, arises from the inner surface of the aponeurosis, external to the beautiful tendon of the temporalis. These fibres, generally slighted or overlooked in the description, and almost universally cut away in the demonstration of this muscle, serve the purpose of aiding the aponeurosis to resist the swelling of the muscle, by contracting from its internal surface towards the tendon or the skull, at the same moment that the powerful part of the muscle is contracting and swelling outward against the aponeurosis. The aponeurosis of the palm serves a very important purpose, not only by strengthening the connexion of the bony structure, but by binding down all the tendons flexing the fingers, and compressing the interosseous and other palmar muscles situate beneath it. The plantar aponeurosis bears a very striking resemblance to the fascia lata in its structure, and relation to the muscles, and to the temporal aponeurosis in the manner of giving origin to muscular fibres. It forms three grand divisions,—in the first place, to embrace the central, external, and internal muscles of the sole; and, on the inner surface of the central portion, the flexor brevis digitorum pedis derives a very considerable part of its origin.

If the coverings of the scapular muscles were not to be traced continuously with the brachial fascia, we should be disposed to class them with the temporal, plantar, and palmar aponeurosis, as they are in various circumstances analogous to them, being much thickened by successive additions of tendinous fibres, and because the infraspinatus has a series of modifying muscular fibres arising from the inner surface of the fascia. This attachment of muscular fibres is very different from what we observe in the origin of the muscles of the fore-arm and leg, where a part of the main body of the muscle arises from the fascia. In the case of the temporalis and infraspinatus, it is a layer of fibres distinct from, and external to, the common mass of the muscle, and serving the purpose heretofore specified.

2d. ***Position, relation of fibres to tendons, &c.***—The effect of position as a modifier of action, may be very fairly illustrated by the origins of the flexor longus pollicis, and the flexor longus digitorum pedis. The long flexor of the great toe, arising from the posterior and inner part of the fibula, and, passing under the inner ankle to its insertion, flexes the great toe in a line corresponding with the inner edge of the foot, or a line drawn from the under surface of the toe to near the centre of the heel. The long flexor of the toes, on the contrary, rising from the outer and back part of the tibia, and running to be inserted in the smaller toes, contracts so as to draw them inwards, or towards a line obliquely crossing the sole of the foot from the outer to the inner side. It would be natural enough for one who was unacquainted with the structure, to expect that the common flexor of the toes should rise from the bone most immediately in a line with the toes to be flexed; that the flexor of the great toe should come from the tibia, and not from the fibula; and the contrary of the common flexor. We shall hereafter see some additional modifying circumstances connected with these two muscles, of great importance and beauty. An instance of a corresponding arrangement may be observed in the relative positions and actions of the extensor longus, and the extensor brevis digitorum pedis. Other instances of the adaptation of situation to the direction of action, will present themselves to any one engaged in studying the subject.

The relation of muscular fibres to the tendons through which they are to act, is another admirable provision for the modification, or rather the direction of their action. This is beautifully seen in all the penniform muscles, especially those belonging to the motions of the foot. They arise by narrow origins, and their fibres run obliquely outwards to receive a tendon on the edge of the muscle, and not at the inferior extremity. Hence, as the successive portions of these muscles come into action, the motion of the foot is effected, and the whole tendon is more and more closely drawn in towards the bones. From the very nature of the space the tendons of the upper part of the foot have to traverse, they could not under any other circumstances act to advantage, although they were furnished with fasciæ and annular ligaments. Another excellent instance of modification, owing to the relation of fibre to tendon, may be observed in the semi-membranosus, one of the great flexors of the leg on the thigh. The origin of this muscle from the upper and posterior part of the tuber ischii, is a broad flat tendon, lying between the biceps and semi-tendinosus. As it is passing through the thicker part of the thigh, this flat tendon has the fleshy fibres attached to it, beginning by short fibres running obliquely, gradually growing thicker and longer for a few inches; then shortening again, with the same obliquity of fibre, the muscle receives the terminal broad tendon, which is to be inserted into the inner and back part of the head of the tibia. Hence this muscle is able to co-operate in the flexion of the leg on the thigh, bringing it directly backwards, and at the same time, by its figure, aiding in giving symmetry to the thigh; whereas, if its fleshy fibres were direct, or corresponding to the course of its origin and insertion, it could do neither.

3d. *Modifying muscles.*—This is a very extensive source of modification to muscular action, and the design of the modifying portions is unequivocally evident. We shall select a few of the most obvious instances as sufficient for the present. The long flexor of the thumb arises on the upper part of the radius below its tuber, and for a considerable distance along that bone towards the wrist. The fibres are necessarily penniform, and the tendon received on the outer edge of the muscle. According to what we have observed on the penniform muscles, this arrangement will draw the tendon more immediately towards the bone; and, if this arrangement were the whole of the muscle, the flexion of the thumb could not take place as advantageously as it now does. But a modifying muscle, having direct fibres, and terminating in a distinct tendon acted on by all its fibres at once, arises from the internal condyle of the humerus, and is fixed into the commencement of the tendon belonging to the radial or penniform portion; and, as its origin is much more favourable to the proper flexion of the thumb, it modifies the action of the lower part of the muscle. It may be said that this modifying portion is not always present: this may be said of various parts whose uses are unequivocal; but this part of the muscle is not frequently absent, perhaps once in ten times, if so often.

Another and more striking instance of modifying muscle is found in the second head of the biceps flexor cruris, the only muscle inserted into the fibula for the flexion of the leg on the thigh. This biceps derives its principal origin from the tuber ischii, in immediate company with the semi-tendinosus, which goes to the inside of the leg. Whoever examines the origin of the biceps, and observes the obliquity of this first head, compared with its insertion, will see that, if this greater part of the muscle were alone, it would rather pull the leg towards the inside, like the semi-tendinosus, than towards the outside. But a second portion of muscle comes off from the outer part of the posterior surface of the thigh-bone, beginning below the insertion of the glutæus maximus into the rough line. This second head has its fibres running obliquely outwards and downwards, and it lays hold of the proper tendon of the biceps on the inside. When the larger portion of the muscle contracts, this short head operates on the tendon, drawing it in the immediate line of the bone; thus correcting the obliquity of flexion which would be produced, if the upper portion coming from the tuber were to act alone.

A modifying structure, having considerable analogy with this, exists in the relation of the gastrocnemius and soleus. The gastrocnemius, arising from the condyles of the femur, is nearly immediately in a line with the os calcis, and, by the projection of the condyles, has great power in commencing the extension of the foot on the leg, though it could not, from the very circumstance of the slenderness of its origin, suffice to sustain much of the weight of the body. The soleus, arising from the head of the fibula and posterior and upper part of the middle of the tibia, and acting on the common tendon fixed to the os calcis, completes the action, draws the heel directly upwards and inwards in a line with the bones; and thus the whole muscle is enabled to sustain a great weight.

The last instance I shall mention of modifying muscle, is the accessory of Sylvius, in the sole of the foot. The situation of the long flexor of the toes has already been referred to, and it has been stated that the object of the flexion is to bring the toes downwards and inwards; but, in passing under the os calcis from the posterior part of the tibia, the tendon passes rather obliquely across the sole, and would draw the toes too directly or violently inwards. This evil is prevented by the intervention of a small but strong mass of flesh, arising from the sinuosity on the inside of the os calcis, and terminating by an oblique insertion into the tendon of the long flexor, just where it separates into four tendons for the lesser toes. This accessory muscle, contracting in the direction of a straight line drawn through the middle of the sole, at the time when the long flexor tendon is drawn immediately inwards, produces that modification of action which is intermediate to what either portion would separately produce.

4th. *Modifying tendinous connexions.*—These will be sufficiently obvious to every anatomist: it will be enough to refer to a few of them. Among the most important may be mentioned the splitting of the tendon of the obliquus internus abdominis, which, with the external, oblique and transversalis, constitutes the sheath of the rectus abdominis. This muscle, in consequence, has its power of flexing the trunk vastly increased, inasmuch as it is affected by every degree of contraction which occurs in the other abdominal muscles, at the same time that its own contractions are performed. In addition, this muscle is broken into portions by tendinous matter, which gives it something of the character of several distinct muscles.

There is a strong tendinous connexion existing between the tendon of the long flexor of the great toe, and the tendon of the long flexor of all the other toes. This tendon enables the flexor of the great toe to participate in the modifying influence exerted by the accessory muscle of Sylvius, recently mentioned under the third head. Various other instances will be recollected, more or less analogous to these.

5th. *Special modifying constructions, &c.*—The most beautiful of these are the trochlea in the orbit of the eye for the obliquus superior, and the hook on the internal pterygoid plate of the sphenoid bone for the circumflexus palati; both of which are so obvious, as to need nothing beyond a mention. The interposition of the patella, by which a pulley is formed at the knee-joint, and of the sesamoid bones, occurring in the tendons of the short flexors of the thumbs and great toes, are also well known.

The annular ligaments of the wrist and ankle are also peculiarly worthy of attention, as modifiers of muscular action, and without which our present construction of muscles would be almost useless. If any one wishes to ascertain how far these instruments direct the action of muscles, let him cut them through, and he will at once see, from the starting forwards of the tendons, that, without the aid of these annular ligaments, the motions of the extremities could not be properly effected. The same principle is resorted to by nature for confining the tendons of the fingers in place, only that in this case the material used is much stronger than that of common annular ligament.

I have not leisure to pursue these investigations further at this time, and am conscious of their manifold imperfections; yet I hope that these remarks may not prove uninteresting to those who are engaged in the study of anatomy, because they appear to me to have a very useful bearing on the physiology and pathology of the muscular system. Some happier genius, by a more extended inspection of all the existing relations of the muscles, may be enabled to explain very many circumstances which now appear dark and difficult, concerning their functions.

ART. II.—***On Periodicity in the Actions of the Animal Economy during Health and Disease.*** By JOHN BELL, M.D. [From the ***Philadelphia Journal,*** No. 2, New Series.]

***Of the Morning.***—WE know that most diseases remit in their violence in the morning, agreeable to the axiom, "Levato sole, levatur morbus." This remission is so marked, that, in mucous and intermittent fevers, especially the tertian and double tertian, persons, in agony during the night, have got up at sunrise, and regained strength enough to go about. Insensible perspiration is besides abundant, and brings great relief: hence dropsical effusions and œdema of the legs are smaller. The hectic fever ceases at this period alone of the twenty-hours. The nocturnal repose of the functions of the nervous system, brings likewise a remission in most spasmodic diseases. The greater number of the phlegmasiæ of the mucous membrane, such as catarrh, croup, &c. are also diminished in violence at this time. In fine, there is scarcely a malady which has an exacerbation in the evening, that has not an intermission in the morning. Asthenic diseases even are milder, on account of the organisation gaining more energy at this epoch.

But this same morning vigour becomes the cause of the invasion of several sthenic diseases. In general, anginæ, vernal quotidian or tertian fever, and simple synocha, are evidently aggravated in the first hours of the day. Ophthalmiæ are more acute; and the hæmoptysis of young persons more readily comes on at this time; as also the sweat of phthisical patients, hysteric swellings, and worms, (no doubt on account of the emptiness of the intestines,) and pyrosis, or water-brash. In fevers called malignant, especially in typhus, there are two exacerbations daily, but that of the morning is, according to Hufeland, more violent than the evening one. So also wounds, ulcers with gangrene, carcinomas, and phlegmons, receive an augmentation of heat, pain, and tension, on the return of the sun above the horizon.

***Of Mid-day.***—In proportion as the sun's elevation increases, bilious fevers, strong nervous emotions, as in mania and hydrophobia, become aggravated. Sauvages has called ***solar mania*** one of those affections which only came on in the heat of the day, and which entirely disappeared at night. He also mentions a woman who became partially deranged precisely at one hour after noon. The phrenitic become violent towards two or three o'clock in the afternoon, with chills and remarkable exacerbations. Musgrave cites an acute arthritic cephalalgia which came on daily at twelve o'clock, and Sauvages furnishes us with an analogous case. We generally find the eruption of the distinct small-pox to commence at this hour. Coma vigil, typhomania, calenture, the violent delirium of the complicated remittent fevers of warm climates, causus, tetanus, and trismus, erysipelas sun-stroke, are at times manifest before noon; but always much more during the heat of the day than at other times. Cholera morbus, spasmodic vomiting, colic, volvulus, and many gastric tertian fevers, have their paroxysms at noon. Finally, hepatitis, gastritis, the bilious diarrhœas of summer in men of an irritable habit, are more especially augmented towards the middle of the day.

*Of Evening.*—It was long ago remarked by Fernel, that all quartan fevers came on after noon, quotidians at early morn, and tertians towards mid-day. But it is more particularly in the evening that the paroxysms of a great number of diseases are produced. All catarrhal affections, heavy phlegmonous pains, inflammation of the organs of animal life, or of relation, are astonishingly aggravated in the evening; owing, no doubt, to the debility of this external life. So also cephalalgia, or sick headaches, are then increased; and comatose affections and apoplexies only strike in the evening or at night. Paralysis, lethargy, syncope, sudden attacks of hypochondriasis and hysteria, slow nervous fever, the oppression from dropsy, articular and rheumatic pains, are necessarily aggravated by this debilitation of the sensitive life. An intermittent febrile hemiplegia has been known to commence at four o'clock in the afternoon, and to cease at six in the morning, and to be cured by the bark; and a periodical cough, beginning at seven in the evening, cut short by opium. It is especially in the evening that suppurative fever comes on with the wounded. Jactation, restlessness, are peculiar to a number of nervous lesions at this period. When hæmorrhages, such as epistaxis, hæmorrhoidal discharges, come on at these hours, they are almost always the result of a spasm, which, without doubt, causes that insupportable anxiety experienced by consumptive persons who have a vomica. Cutaneous diseases, itch, herpes, chilblains, are more troublesome in the evening.

It might at first be thought that the fatigues of the day, the exercise of the senses, the introduction of fresh chyle into the blood from the nutriment previously taken, and even the irritation from remedies, dispose the animal economy to a general exacerbative movement: but, in reply to this, it may be alleged that, even though the invalid sleep through the day, and adhere to a most rigorous regimen, hectic fever, for example, will come on at the accustomed hour. We find, on the other hand, that diseases of the throat, and some other matinal affections, are dissipated in the evening.

In general, the disorders of the sub-diaphragmatic organs in old men, such as those of the urinary passages, hæmorrhoids, gout, melancholy, dysentery, enlargements and obstructions of the viscera, as of the spleen and mesentery, are more particularly aggravated at this epoch.

*Of the Influence of Night.*—It is well known that sthenic affections, exacerbating in the day, have a remission in the humidity, cold, and obscurity of the night; and that, on the other hand, the diseases which are milder during the day, such as fevers, mucous phlegmasiæ, catarrhs, croup, affections of the lymphatic system, dropsy, cachexia, asthenic complaints, and paralyses in general, are aggravated in the night. There are, however, certain states of our organs at particular hours, independent of the sensible properties of the atmosphere, or of light and darkness; and hence, as remarked by an ingenious writer,* to whom I am largely indebted on this occasion, the human body is, in this

* Virey.

respect, like a living clock, wound up by nature, and kept a-going by the rapid movements of our planet and the sun.

Humboldt tells us of a certain countess at Madrid, who lost her voice at sunset, and only recovered it at early dawn. This paralysis of the recurrents of the eighth pair disappeared in the climate of Naples, and reappeared in that of Rome. Other nocturnal paralyses, deliriums, and vertigos, have been observed to come on at the same epoch, which renders probable what Aristotle relates of a tavern-keeper at Tarentum, who was very rational during the day, but became insane regularly on the approach of night. The hemeralopses at this time experience very sensibly that singular collapse which prevents them from seeing; while those labouring under nyctalopia, on the contrary, see better in a feeble light: and we find at this period some headaches begin, and others cease. "A woman," says Baillou, "fell into a state of insensibility at sunset, and recovered her vigour in the morning."

After this general view, it becomes us to notice the phenomena observable in the several hours, or stated periods of the night. The oppression of incubus, for example, only comes on in the first sleep; when also the suffocating feeling from ascites is most distressing. It is the same with the venereal pains of the bones, rheumatism, and scurvy. Asturian, leprosy, croup, catarrhal affections, hooping-cough, and periodical cough, are greatly aggravated during this same period. It is then also that gangrena senilis, passive hæmorrhages (so called), petechiæ, and the danger from adynamic or low fevers, and contagious and pestilential ones, are increased, on account of the general depression in the animal economy.

Towards two or three o'clock in the morning, when the pulse rises after the first sleep, another order of actions begin. Sydenham was astonished to find the gout make its attack so exactly at this time. Floyer makes a similar remark on asthma. Dropsical spasms, violent palpitations, which awake the alarmed hypochondriacal, or frightful dreams, are then experienced: it is then that somnambulists rise and move about; whilst the wakefulness of old men, and that produced by slow fever, come on. The greater number of epilepsies, the paroxysms of which supervene in the night, declare themselves at this period.

After this state of spasm, as it may be termed, there is an excitation not less remarkable. The consumptive then have sweats; gastritis and hectic fevers are exacerbated; the apthæ and miliary eruptions of children then effloresce; the critical sweats in mucous fevers, different eruptions, tinea, and others, begin, or are augmented. The dyspeptic invalid, who awakes perchance at midnight, or a short time after, and feels his tongue and mouth moist, must be often surprised at the change in his feelings at early morn, when the same parts are rough, parched, and loaded. The most salutary critical perturbations are then brought about; for they are prepared by sleep, which leads to a morning remission. Winter, old age, lymphatic temperaments, are aggravating circumstances in nocturnal affections.

Let us conclude these remarks on the nocturnal period, by repeating the observations of Ramazzini, Home, and Pringle. The first tells us of an epidemic disease in 1691, the symptoms of which became alarming after sunset, to such a degree, that the sick were in a state of extreme depression, and almost dying during the night. The two last-named writers have transmitted to us the history of an epidemic remittent fever which prevailed among the English troops in Flanders, in 1743, and which presented the same characters. There was a regular and almost entire remission of the symptoms during the day; no complaints made; the pulse not much accelerated: but, on the approach of night, uniformly and without any sensation of coldness, the fever was aggravated, and the symptoms became so intense, that the patient was often delirious. In the morning, again, the pulse did not indicate the danger which was just escaped.

As respects the proportionate mortality in the diurnal revolution, Virey tells us, in his Thesis, that the greatest number of deaths happens at early morn, rather after than before sunrise; that there are more in the day than in the night, by a sixth, and that, in the evening, the greatest mortality is on the approach of night. More than one-half die at different hours, according to the season, and the rising or setting of the sun. In summer, in the months of June, July, and August, the mortality is sensibly increased towards two or three o'clock in the afternoon; owing doubtless to the heat, since this epoch is healthy in the cooler months.

The periods in which the fewest deaths occur, are from ten o'clock at night till three in the morning, corresponding to that of the first sleep; as also those from eight to ten in the morning, and from twelve to one in the day. It would follow from this, that the inauspicious hours (*horæ infaustæ*) for the sick, must be also the least favourable to persons in health; the same causes acting, we presume, on all. It is probable, moreover, that, as nature determines favourably or unfavourably by the periodical returns, which we have seen correspond with certain hours in the day, people are most liable to perish at the hour when the disorders with which they are attacked has its exacerbation.

The inferences to be drawn from this knowledge of the periodicity of the functions of the animal economy in health and disease, are such as to admit of the greatest variety of application in hygiene, pathology, and therapeutics. We have seen that each period of the diurnal revolution has a peculiar character, marked by the excitation of a system of organs in relation with it.

Sleep during the night, wakefulness in the day, an active matinal life, relaxations in the evening; such is the natural order of things, and the man of nature, the child, and the countryman, who are obedient to these instinctive impulsions, commonly enjoy vigorous health. They who have been enervated and enfeebled by long watching, exercises, and exciting pleasures, prolonged through the evening on to the midnight hour, can only hope for a restoration of health, juvenile strength, and mental vivacity, by retracing their steps, and following the course pointed out by nature, and sanctioned by the experience of all ages and nations. The old man who sleeps but little, and the dissipated and indolent one who sleeps long in the morning, dislike retiring to rest until the night is somewhat advanced; while the child, who goes to sleep early, awakes at the dawn. The former accelerate the approach of the evening and decay of life by such means; the last keeps up the freshness and beauty of its morn.

The hour of repast cannot be a matter of indifference, and ought to be regulated by the periods of the night and day, as well as by the pursuits, age, and constitution of the individual. It is known that nutriment taken in the evening produces an accumulation of mucosities or glairy juices in the intestinal canal. If, then, we may presume that other periods make other humours predominate by digestion, (bile, for example, taking the ascendency in the heat of the day,) the morning repast will be the most salutary and renovating. Would it not be better for the old man to take his meal in the morning, and the one of a lymphatic constitution in the heat of the day? Our advice on such subjects must, however, be modified by the condition of the digestive organs: thus, in the states already mentioned of morning debility of these parts, some time might be allowed to elapse after rising, and a tonic mixture or infusion drank, to produce a slight reaction before food is taken. So also in chronic periodical affections, which correspond with the physiological excitations in the diurnal cycle, we might hope to remove them by inverting the usual order in the habits of the individual, in reference to diet, exercise, and even sleep. This means of relief now suggested has been too much overlooked in the treatment of chronic diseases, which are assailed in a careless manner, without physiological guidance. The difference in the hours of attack of any disease will materially affect its diagnosis by the intelligent physician. An epilepsy, for example, the paroxysms of which come on constantly in the evening, will be found to have a different cause from the epilepsy habitually coming on in the morning, and would indicate a different method of treatment; the first being probably more nervous than the second.

We ought also to inquire whether there does not exist a certain affinity between night and cerebral affections, by which their violence is augmented; and, if the diseases of the thorax are not aggravated in the morning, and those of the stomach, liver, and spleen, governed by the heat of the day; and the disorders of the hypogastric viscera by the evening, as many facts would seem to announce.

How vitally important is the study of those opportune pauses, or remissions and intermissions, which must often be taken advantage of at the very hour, as in periodical fevers; for, if the occasion be once missed, it may never again return, and the patient will be carried off in the next paroxysm. The same advantages attend a careful observation of the daily periods of many other desolating diseases, so as to learn their tendency, returns, critical perturbations, the precise hour of hemorrhages, evacuations by sweat, urine, stool, expectoration, &c. If we neglect these circumstances, we know not the time when a remedy can be given with advantage, and may as often increase the disturbance in the animal economy as tranquillise it. By knowing whether diseases, such as some contagious ones, pursue a determined course, and have a certain duration, in despite of all therapeutical agents, we remain satisfied with moderating the more violent symptoms, preventing the disorganisation of parts or new formations, without sinking the system too low for the performance of its necessary functions. On the other hand, in diseases of habit, as in prolonged intermittent fevers, some epilepsies, various nervous affections, we attempt the revolutionising or perturbating plan, to break up the diseased associations. We are at the present time unhappily too artificial in our views of disease. If we bring our therapeutical phalanx into action, it is all we ask; forgetting that we may prostrate without destroying, and drive away the enemy from its lodgment, only that it may take another still more disadvantageous to us. If we are to be the servants of nature in the cure of disease, it behoves us to watch with assiduity its augmentation and decline, its exacerbation and remission, and to distinguish its natural periods from those caused by our own presumptuous haste or ignorance. This is a copious theme, on which, perhaps, I may hereafter be tempted to dilate.

Every medicine is not equally indicated at every hour; a truth not sufficiently dwelt on by writers and teachers of materia medica. Thus, narcotics, sedatives, &c. would, except in extreme cases, be misplaced in the morning, when all the faculties tend to wakefulness; but these same remedies will have a more profound and salutary action in the evening, because the powers of nature then tend to repose and sleep. Hence Sydenham always prescribed an opiate on the evening of the day when he had administered an emetic or a purge; and this practice is very generally imitated, to calm irritation.

The morning remission is generally the chosen time for the greater number of evacuants and alteratives, vermifuges, astringents, and tonics, as the first passages are then most empty of alimentary substances.

Baths, lotions, emollients, and cooling remedies, have a better effect after the great heat of the day on the muscular, fibrous, and nervous systems: hence we find that the ancients used the bath after their *cena*, or supper. Bleeding or depletion of the venous system, especially if there is a threatened congestion of the brain, is better in the evening. But, if there be depression or prostration of power, as in low fevers, we must, as the night advances, apply blisters and rubefacients, and urge the use of stimulating and cordial remedies, so as to keep up the action of the system until after midnight, and carry thereby the patient through the critical hours immediately succeeding it, as it is then we most frequently see him begin to sink, and, if unaided, he becomes more and more depressed until morning, when death closes the scene.

